# Surface Curvature Differentially Regulates Stem Cell Migration and Differentiation via Altered Attachment Morphology and Nuclear Deformation

**DOI:** 10.1002/advs.201600347

**Published:** 2016-12-20

**Authors:** Maike Werner, Sébastien B. G. Blanquer, Suvi P. Haimi, Gabriela Korus, John W. C. Dunlop, Georg N. Duda, Dirk. W. Grijpma, Ansgar Petersen

**Affiliations:** ^1^ Julius Wolff InstituteCharité—Universitätsmedizin BerlinAugustenburger Platz 1D‐13353BerlinGermany; ^2^ Department of Biomaterials Science and TechnologyTwente UniversityP.O. Box 2177500AEEnschedeThe Netherlands; ^3^ Department of Biomedical EngineeringEindhoven University of TechnologyP.O. Box 5135600MBEindhovenThe Netherlands; ^4^ Department of Oral and Maxillofacial DiseasesUniversity of HelsinkiHaartmaninkatu 8FI00014HelsinkiFinland; ^5^ Max Planck Institute of Colloids and InterfacesAm Mühlenberg 114476PotsdamGermany; ^6^ Berlin‐Brandenburg Center for Regenerative TherapiesCharité—Universitätsmedizin BerlinAugustenburger Platz 1D‐13353BerlinGermany; ^7^ Department of Biomedical EngineeringUniversity Medical Centre GroningenUniversity of GroningenAntonius Deusinglaan 19713AVGroningenThe Netherlands

**Keywords:** 3D surface curvature, cell migration, nuclear mechanics, osteogenic differentiation, stereolithography

## Abstract

Signals from the microenvironment around a cell are known to influence cell behavior. Material properties, such as biochemical composition and substrate stiffness, are today accepted as significant regulators of stem cell fate. The knowledge of how cell behavior is influenced by 3D geometric cues is, however, strongly limited despite its potential relevance for the understanding of tissue regenerative processes and the design of biomaterials. Here, the role of surface curvature on the migratory and differentiation behavior of human mesenchymal stem cells (hMSCs) has been investigated on 3D surfaces with well‐defined geometric features produced by stereolithography. Time lapse microscopy reveals a significant increase of cell migration speed on concave spherical compared to convex spherical structures and flat surfaces resulting from an upward‐lift of the cell body due to cytoskeletal forces. On convex surfaces, cytoskeletal forces lead to substantial nuclear deformation, increase lamin‐A levels and promote osteogenic differentiation. The findings of this study demonstrate a so far missing link between 3D surface curvature and hMSC behavior. This will not only help to better understand the role of extracellular matrix architecture in health and disease but also give new insights in how 3D geometries can be used as a cell‐instructive material parameter in the field of biomaterial‐guided tissue regeneration.

## 1 Introduction

In native tissues, cells reside in an extracellular matrix (ECM) which regulates and guides multicellular organization. Cells have the ability to sense physical properties of the ECM and respond to them.^1^, ^2^ The ECM therefore plays an important role not only in the maintenance of the structural integrity of tissues but equally during tissue regeneration where the interplay between cells and ECM is altered as a consequence of injury.^3^, ^4^ In regeneration, human mesenchymal stem cells (hMSCs) from the bone marrow stroma are believed to play an important role due to their ability for self‐renewal and differentiation into different mature cells like osteoblasts, adipocytes and chondrocytes.^5^ Numerous studies have shown that the mechanical properties of the cell's microenvironment, such as the substrate stiffness, have a fundamental effect on hMSC cell fate and function and impact tissue regeneration.^6^, ^7^, ^8^, ^9^ Recently, evidence is rising that the geometrical properties of the cell's environment also play an important role as regulators of cell behavior.^10^, ^11^, ^12^, ^13^, ^14^, ^15^, ^16^, ^17^, ^18^, ^19^ Using geometric features, such as pore geometry, as a cue to direct tissue regeneration is compelling since it may allow a biomaterial to steer cell function purely by its shape and hereby contribute to tissue regeneration. Although geometry guided cell behavior has been studied extensively on 2D substrates, the knowledge of how 3D geometry affects cell behavior is strongly limited. A deeper insight into the role of geometrical cues on cell functions, such as cell migration and differentiation, is therefore needed for the design of 3D biomaterial environments fostering tissue regeneration.

In earlier studies, micropatterned substrates with various geometries have been used to investigate the relevance of cell shape for migration and differentiation. It was found that substrate geometries can affect stress fiber‐ and focal adhesion organization in 2D.^20^ Furthermore, tensional stress concentration at corner regions of square adhesive islands promoted lamellipodia extension.^21^ Micropatterned adhesive islands were also used to control the degree of cell spreading and it was demonstrated that increased spreading led to osteogenic differentiation, while a more round cell morphology promoted adipogenesis.^22^ In addition, cells on adhesive islands of various geometries but with constant area were shown to have different differentiation profiles. Shapes with a high aspect ratio and high subcellular curvature promoted increased cellular contractility that led to osteogenic differentiation.^10^

In recent years, there is increasing evidence that 3D substrate geometry is a further relevant material parameter influencing tissue growth and organization. Using biomaterials with macropores of various shapes, it has been demonstrated in the context of bone healing that the local tissue growth rate is strongly influenced by curvature.^12^ Preferential tissue growth was observed in concave areas and at high curvature magnitude while tissue growth on uncurved and convex regions was minimal.^12^, ^23^ Curvature driven cell organization in macroporous biomaterials was also shown to influence ECM distribution.^24^ Furthermore, there are first indications that also single cell behavior is influenced by 3D surface curvature as cells were observed to actively migrate out of concave pits, while cells attached and proliferated on convex structures.^11^ Recently, it was shown that the diameter of 3D spherical pores in scaffold structures has an impact on cell morphology and osteogenic differentiation of MSCs.^15^ Together, these studies give the first evidence that cell behavior and organization are altered by curvatures much larger than the cell size. However, an understanding of how the macrocurvature of the surface (in a range larger than cell size, i.e., hundreds of micrometers) is sensed by cells and influences their function is missing even though it is essential in exploring surface geometry as a cell‐instructive material parameter. A systematic study of the cell interaction with controlled substrate curvatures on a single‐cell‐level is lacking.

Here, we studied the influence of well‐defined concave spherical, convex spherical and flat structures on the migratory and differentiation behavior of hMSCs with relevance for bone tissue regeneration. To do this, we developed 3D macrotopographic cell culture chips based on stereolithography. We demonstrate that concave substrates promote faster cell migration, while convex substrates induce osteogenic differentiation. Furthermore, we suggest a mechanism for the observed curvature dependent alterations in cell behavior based on cytoskeletal force‐driven modulation of cell and nucleus geometry.

## 2 Results and Discussion

### 2.1 Concave and Convex Surfaces Induce Different hMSC Migration Modes

Cell culture substrates were designed containing convex and concave spherical structures with diameters ranging from 250 µm (principle curvature (κ) = 1/125 µm^−1^), which is 2–3 times the size of hMSCs in a spread state, to 750 µm (κ = 1/375 µm^−1^), notably larger than cell size. Cell culture chips were produced by stereolithography using a poly(trimethylene carbonate) (PTMC)‐based resin. This method was chosen since it permits the fabrication of versatile 3D structures for the future implementation into more complex biomaterial designs.^25^

**Figure 1:**
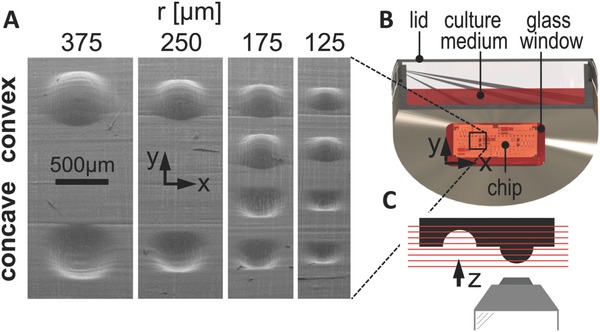
Setup used to investigate curvature‐dependent cell behavior. A) Scanning electron microscopy image of the cell culture chip showing flat, convex, and concave spherical surfaces. B) Experimental microscope setup for time lapse and immunohistochemistry imaging. The chip was placed on the glass window of a custom‐made culture dish with the structures facing downward. C) 3D image stacks were recorded using an inverted confocal/multiphoton microscope.

**Figure 2:**
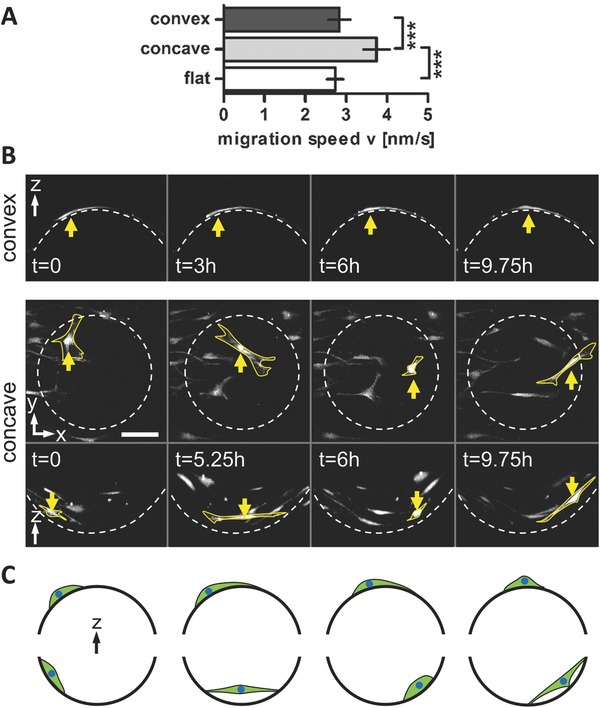
hMSC migration speed is increased on concave compared to flat and convex surfaces. A) Quantification of hMSC migration speed on flat, concave and convex surfaces over 24 h shown as mean ± 95% confidence interval. ***P < 0.001. B) Projections of subsequent *z*‐stack images of a time lapse recording of migrating cells on a convex spherical structure (top) and in a concave spherical structure (bottom) with κ = 1/175 µm^−1^ in top view and side view. The yellow arrow highlights a cell showing a typical migration mode on convex or concave structures, respectively. Note the flat cell morphology on convex structures and the slow but constant displacement, while in concave structures cells form long and thin extensions over the concave pit followed by a large displacement of the cell body. Scale bar 100 µm. C) Schematic representation of the observed migration regimes of cells in convex (top) and concave (bottom) spherical structures. Cells on convex structures showed a typical 2D MSC migration behavior, while cells on concave surfaces adapted a spider‐like conformation which is characterized by long cell extensions spanning over a large free space followed by fast cell body displacement.

Stereolithography was proven to be suitable for the production of 3D substrates with specific geometrical features, confirmed by scanning electron microscopy images (**Figure** **1**A). Substrates were homogeneously seeded with fluorescently stained bone‐marrow derived hMSCs and cell migration was observed for up to 24 h using time lapse multiphoton microscopy (Figure 1B,C). According to microscopic evaluation, hMSCs were distributed evenly on the PTMC chip surface without showing regions of cell accumulation. The mean migration speed of the individually tracked cells on the different structures is shown in **Figure** **2**A. Cell migration speed on concave spherical surfaces (${\bar{v}}_{\mathrm{cell}}\;=\;3.7\;\pm \;0.2\;\mathrm{nm}\;s^{-1}$) was significantly higher than on convex (${\bar{v}}_{\mathrm{cell}}\;=\;2.8\;\pm \;0.3\;\mathrm{nm}\;s^{-1}$) structures and flat surfaces (${\bar{v}}_{\mathrm{cell}}\;=\;2.7\;\pm \;0.3\;\mathrm{nm}\;s^{-1}$) (Figure 2A). No significant difference was found between different curvature magnitudes (Figure S1, Supporting Information).

These results are in agreement with a study of Park et al., who showed that mouse fibroblasts move significantly faster in concave pits with a diameter of 200 µm and a depth of 50 µm compared to flat surfaces.^11^ In further agreement with our findings, cell speed on convex posts was not significantly different from flat surfaces. In the mentioned study however, both the time lapse experiments and the immunohistological data were recorded as 2D images. Therefore, information about the details of cell movement and attachment was missing. In our study, *z*‐stack time lapse recordings of migrating cells provided 3D information on the cellular attachment‐morphology during migration on the differently curved surfaces. This allowed us to gain a better understanding in the mechanism that causes the significant difference in migration speed between concave and convex substrates. The time lapse sequences revealed distinct migration regimes on convex and concave structures. Cells on convex structures showed a typical 2D MSC migration behavior; protrusion of the leading edge, cell body translocation and retraction of the rear.^26^ The constant repetition of this migration cycle results in a relatively constant migration speed over time. In contrast, cells on concave structures formed long cell body extensions reaching over a large free spanning distance and attached at a small region far from the center of the cell (Figure 2B,C). A closer look at subsequent time frames revealed a two‐phase extend‐and‐pull movement: 1) the formation of a cell body extension with a remarkable increase in length spanning over the concave pit and rather slow movement of the cell body and 2) a pull of the cell body over the pit toward the adhesion point of the cell extension together with a dramatically increased speed of cell body movement (Figure 2B and Movie S1, Supporting Information). This remarkable adhesion and translocation strategy resulted in a fast but twitchy cell migration.

### 2.2 Cell‐Substrate Contact Area Explains hMSC Migration Behavior on Curved Surfaces

**Figure 3:**
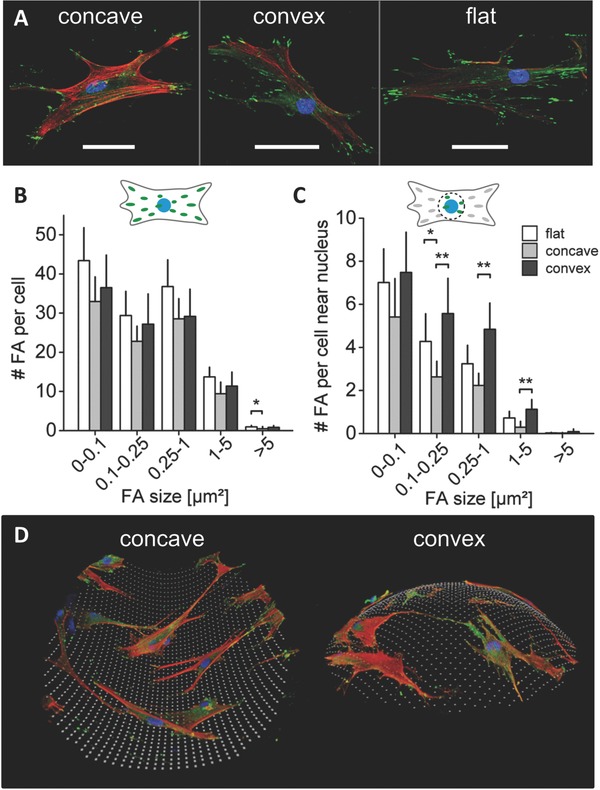
Curvature driven alterations in cell attachment. A) Representative immunohistochemical images of vinculin (green) identifying focal adhesions, F‐actin (red) and nuclei (blue) for hMSCs on concave and convex spherical surfaces (κ = 1/175 µm^−1^), and a flat surface after 2 d in expansion medium. Scale bar 50 µm. B) Number of total amount of focal adhesions (FAs) per cell, divided into different focal adhesion size classes. C) Number of focal adhesions per cell near the nucleus for the same focal adhesion size classes as in (B). No significant differences were found in the total amount and size of focal adhesions per cell on the different structures. However, near the nucleus a significant lower number of focal adhesions (size > 0.1 µm^2^) was found in cells in concave surfaces. Mean ± 95% confidence interval. *P < 0.05, **P < 0.01. D) 3D reconstruction of immunohistological stained cells (F‐actin in red, FAs in green, and nuclei in blue) on a concave and convex surface. Cells on concave surfaces showed an upward stretched cell morphology where a substantial part of the cell body is not attached to the surface. Cells on convex surfaces were fully attached to the surface.

Confocal microscopy of immunohistochemically stained cells allowed us to study cell morphology on the spherical surfaces in more detail and in 3D. Substrate curvature had a strong influence on cell attachment to the surface. In concave spherical structures, the cell body was pulled upward, minimizing the contact area with the substrate. Consequently, concave arc‐like cell contours with strong actin filaments were observed between the well‐separated individual adhesion points (**Figure** **3**A). A similar arced cell contour with strong actin filaments was found in previous studies, where microprinted islands on a 2D surface were used to create specific adhesive and nonadhesive areas to restrict cell adhesion to distinct points.^20^, ^27^ An inwardly curved cell contour was observed between the attachment points which could be explained by a physical model. In this model, surface tension pulls the cell membrane inward to decrease the cell's surface area and the counteracting forces developed by the cytoskeleton maintain the cell shape by exhibiting strong tensioned actin filaments.^27^ It is surprising that we see a similar effect on concave 3D substrates since here, in contrast to distinct adhesive points on a 2D surface, cells had the possibility to adhere to the entire surface area. However, we found that due to the extra dimension, cells on concave spherical surfaces stretched upward in the *z*‐direction in addition to the x‐y‐stretching on flat 2D surfaces. Through this mechanism, the contact area between the cell and the surface was reduced to distinct adhesion points at the periphery of the cell (Figure 3D and Movie S2, Supporting Information). The mean migration speed was higher on concave than on convex curvatures for all studied curvature magnitudes (Figure S1, Supporting Information). The differences between the mean migration speed on convex and concave structures was variable between the different curvature magnitudes, however, no correlation was found between the curvature magnitude and the migration speed. The uplifting cell morphology was observed in the concave spherical pits of all sizes. This suggests that if the concave curvature magnitude exceeds a certain threshold, that is below the smallest curvature of κ = 1/375 µm^−1^ investigated here, the cell reduces the contact area to the substrate and stretches upward. The reduction in cell‐substrate contact area as soon as the cell lifts off the surface explains the limited influence of the curvature magnitude on the migration speed.

Surprisingly, the analysis of vinculin, a focal adhesion protein, showed no significant difference in the total amount and size of focal adhesions per cell on the different structures (Figure 3B). However, in 3D reconstructed confocal images of cells on concave spherical surfaces, focal adhesions were found mostly in the periphery of the cell at the ends of arc‐like actin filaments. The quantification of focal adhesions in the vicinity of the nucleus revealed a significantly lower number of focal adhesions on concave compared to convex and flat surfaces (Figure 3C), confirming that the center of the cell body tends to be detached from concave surfaces. In this morphology, the cell body extensions could move freely and fast over the concave surface by continuous remodeling of the adhesion points associated with the above described increased cell motility (Movie S1, Supporting Information).

Taken together, 3D observations of the cell‐substrate interactions on spherical surfaces revealed a difference in cell attachment on convex and concave surfaces that can explain the distinct cell migration regimes on the curved surfaces. hMSCs on concave surfaces adopt a spider‐like morphology where the cell body is stretched upward, away from the surface, while the extensions (“legs”) at the periphery of the cells remain attached to the walls of the concave surface. In contrast, cells on convex structures adopt a snail‐like configuration with full contact to the substrate (Figure 3D). These observations are in accordance with the chord model presented by Bidan et al., that defines cells as tensile elements that are stretched upward between the focal adhesions on concave surfaces, while being pulled downward toward the surface on convex substrates.^28^ While the cell body on concave surfaces only partially adopted the curvature of the surface by lifting away from it, cells on convex surfaces had to bend and adopt their shape to the curvature of the surface (Figure 3D, and Movies S2 and S3, Supporting Information). Consequently, on the convex surfaces, a larger contact area has to be remodeled for cell movement leading to a slower and more constant migration. On the contrary, the small contact area of the upward lifted cell could explain the twitchy extend‐and‐pull migration regime on concave surfaces.

### 2.3 Convex Spherical Surfaces Induce Osteogenic Differentiation of hMSCs

**Figure 4:**
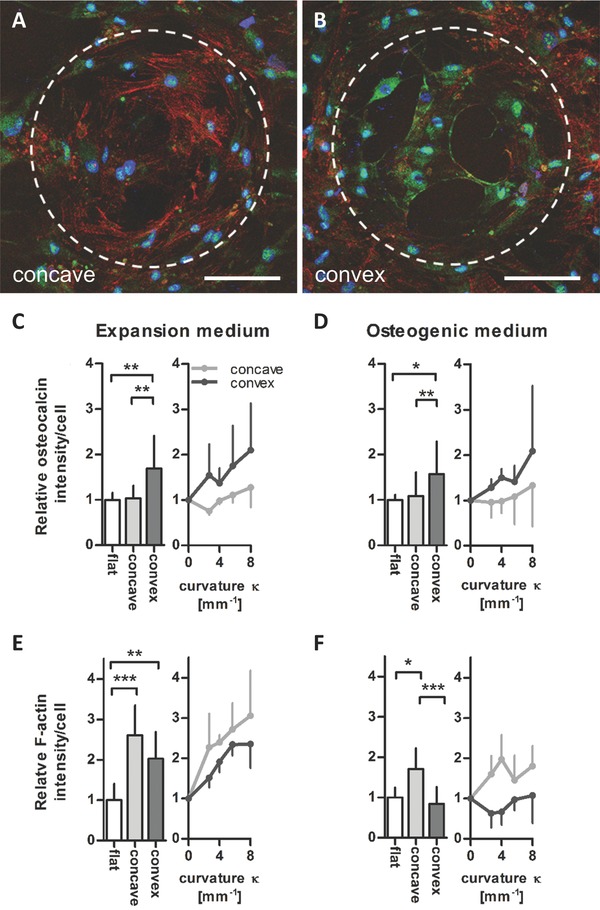
Surface curvature affects the F‐actin cytoskeleton and osteocalcin levels in hMSCs. Representative immunohistochemical images of osteocalcin in hMSCs on A) a concave and B) a convex spherical surface (κ = 1/175 µm^−1^) after 10 d in osteogenic medium (osteocalcin in green, nuclei in blue, and F‐actin in red). Scale bar 100 µm. Dashed lines highlight the contour of the spherical surface. C–D) Quantification of osteocalcin intensity/cell for different curvatures κ (mm^−1^) shown as mean values of all concave/convex surfaces (bar charts) and for the individual curvatures investigated (point charts). After 10 d in C) expansion medium and D) osteogenic medium, significant higher levels on convex spherical surfaces compared to flat and concave surfaces were revealed. E,F) Quantified F‐actin intensity/cell levels were highest on concave spherical surfaces after 10 d in E) expansion medium and F) osteogenic medium. Mean ± standard deviation. *P < 0.05, **P < 0.01, ***P < 0.001.

Next, we asked whether the observed curvature‐induced changes in cellular attachment morphology could also influence osteogenic differentiation. Bone‐specific marker expression was analyzed by immunohistological quantification of osteocalcin in hMSCs on the curved and flat surfaces. Additionally, the F‐actin signal intensity was quantified since the filamentous polymerized actin network is an integral part of the cytoskeletal force generation machinery that is involved in cell shape induced stem cell differentiation.^22^ Cells were cultured on the 3D macrotopographic cell culture chips for 10 d in either expansion medium or osteogenic medium, then fixated and stained for actin filaments, osteocalcin and cell nuclei. As shown in **Figure** **4**A,B, a strong osteocalcin signal was observed on convex structures. Interestingly, only a weak F‐actin intensity was observed in cells on these convex structures, while cells that resided in concave structures showed a strong actin but low osteocalcin signal. This is in contrast to previous studies where a higher cytoskeletal tension, associated with a strong actin cytoskeleton, was shown to promote osteogenic differentiation.^14^, ^22^ The curvature of the transition region from flat to convex surface is highly concave and the region from flat to concave surface strongly convex. Cells therefore show opposing intensity levels in this transition region compared to cells on the spherical surface.

Quantification of the intensity levels confirmed significantly higher levels of osteocalcin in cells cultured on convex spherical structures compared to flat and concave spherical structures, while cells on concave structures showed significantly higher F‐actin levels (Figure 4C–F). In previous studies, 2D adhesive patterns of different sizes, shapes or aspect ratios have been used to demonstrate how stem cell differentiation is influenced by the shape of either individual cells or multicellular structures.^10^, ^14^, ^22^ The results presented here show that 3D substrate geometries of equal size and shape but of opposite curvature (convex vs concave) can also have a significant influence of osteogenic marker expression. Furthermore, the promoting effect of convex curvatures on the osteocalcin intensity was observed both in cells cultured in expansion medium and in osteogenic medium. This is remarkable since it indicates that substrate geometries with features larger than the cell size can promote osteogenic differentiation of hMSCs, even in the absence of osteogenic growth factors. In addition to the difference between convex and concave surfaces, both F‐actin and osteocalcin intensity levels increased with increasing curvature. Although the differences between the curvature magnitudes were not statistically significant, the trend indicates that cells can also sense and respond to the magnitude of curvature.

### 2.4 Convex Surfaces Induce Nuclear Compression and Associated Higher Lamin‐A Levels

**Figure 5:**
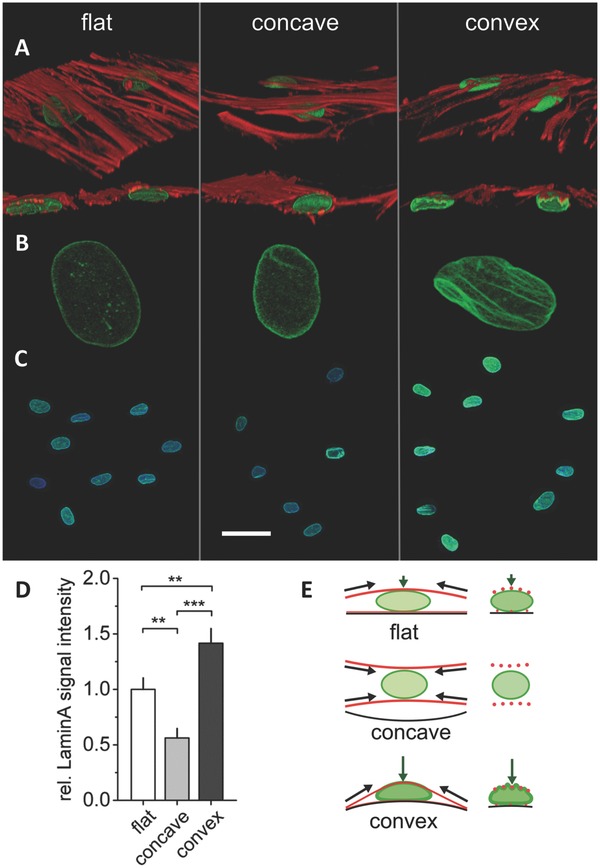
Curvature induced cytoskeletal tension affects nucleus morphology and lamin‐A levels. A–C) Representative immunohistochemical images of lamin‐A (green), F‐actin (red), and nuclei (blue) in hMSCs on concave and convex spherical surfaces (κ = 1/175 µm^−1^) and a flat surface after 10 d culture in expansion medium. A‐B) F‐actin bundles cross over and indent into the nucleus on convex spherical surfaces creating grooves in the nuclear membrane. Cell nuclei were flattened but without grooves on flat surfaces and the nuclei of cells on concave surfaces presented a round morphology. C‐D) Cells on convex structures showed highest lamin‐A levels in immunohistological staining verified by quantification of lamin‐A signal intensity shown as mean ± 95% confidence interval. **P < 0.01, ***P < 0.001. Scale bar in (C) equals to 50 µm. E) Schematic representation of the cytoskeletal forces acting on the nucleus (F‐actin in red, lamin‐A in green). Cytoskeletal tension creates a push force on convex spherical surfaces leading to compression and deformation of the nucleus. A cytoskeletal pull force on concave surfaces (see also Figure 6) leads to a relatively low exposure of the nucleus to cytoskeletal forces resulting in a rounded nucleus shape and low lamin‐A levels.

In previous studies, a larger contact area has been correlated with a higher number of focal adhesions per cell and demonstrated the promotion of osteogenic differentiation via RhoA mediated actin‐myosin generated tension.^22^, ^29^ This, however, does not explain our observations where osteogenic differentiation was higher on convex structures compared to flat and concave structures: The number of focal adhesions per cell did not differ significantly between convex and flat structures (Figure 3B) and the F‐actin signal was even lower on convex compared to concave surfaces (Figure 4E,F). However, we observed that the bent cell morphology on convex structures had a strong effect on the shape of the nucleus. Cell nuclei were flattened and stretched over the convex surface, in some cells even resulting in a bean‐like nuclear morphology. Individual fibers of the perinuclear actin cap applied a sufficiently strong push‐force on the nucleus to indent into the nuclear membrane resulting in a grooved membrane surface (**Figure** **5**A,B). On flat surfaces cell nuclei were flattened but showed a rather smooth membrane while on concave structures no pronounced deformation and almost spheroidal nuclei were observed.

Immunohistochemical staining of lamin‐A was performed to obtain a deeper insight in the mechanical response of the nucleus to this curvature‐driven deformation. Figure 5C shows representative confocal images of lamin‐A expression on flat, concave and convex spherical surfaces. Quantification of signal intensity revealed 2.5× higher lamin‐A levels on convex compared to concave surfaces and 1.4× higher values in cells on convex compared to flat surfaces (Figure 5D). The higher lamin‐A levels and observed indentation of actin stress fibers in the nuclear membrane suggests that high intracellular tensions are exerted on the nuclei of cells on convex surfaces.

Lamins are proteins at the inside of the nuclear membrane that have a substantial influence on the elastic modulus and viscosity of the nucleus.^30^ They are mechanically coupled to the actin stress fibers through linkers of nucleoskeleton and cytoskeleton complexes and have been proposed to be involved in protecting the chromatin cargo of the nucleus against forces acting from outside via the cytoskeleton on the nuclear membrane.^31^, ^32^ Recent studies have provided evidence that actin stress fibers can exert a compressive load on the nucleus on 2D substrates. Cells comprised with a perinuclear actin cap were shown to transmit intracellular tension to the nucleus which provided a driving force for lamin A/C and acetylated histones concentration at the apical side of the nucleus. On the contrary, cells that do not form an actin cap or cells with a disrupted actin cap did not show this spatial polarized distribution of nuclear lamia and transcription‐active domains in the nucleus.^32^ Furthermore, 2D patterned substrates of various aspect ratios were used to observe the effect of cell shape on the interplay between actin cytoskeleton and nucleus. An increase in aspect ratio resulted in the formation of distinct apical stress fibers which indented in the nuclear membrane and not only decreased the nuclear height but also provided stability in nuclear positioning and controlled heterochromatin assembly.^16^ Cells cultured on 2D micropatterns of various shapes, sizes and aspect ratios showed differential changes in gene‐expression profiles, which were preceded by geometry‐dependent changes in nuclear morphology and histone acetylation.^17^ Increased lamin‐A levels were reported in stiff tissues and in cells cultured on stiff artificial substrates and it was shown that this nuclear stress‐protection was associated with an osteogenic cell fate.^30^ Interestingly, a genomics approach study revealed that substrate‐topography related gene regulation tend to occur at the telomeric ends of the chromosomes, where osteogenic genes are clustered, rather than in the centromeric region. Mechanical alterations of the nuclear envelope might thus have a direct mechanical control over osteogenic gene regulation via telomeric chromatin‐lamin interactions.^33^ Together, the results of these studies suggest that substrate stiffness and 2D substrate geometries can affect gene‐expression profiles as a result of increased cytoskeletal tension transmitted toward the nucleus by actin stress fibers.

Our results show that in addition to substrate stiffness and 2D geometry, 3D surface curvature is a further relevant parameter that can change the stress fiber forces on the nucleus, nucleus morphology and associated changes in lamin‐A expression. The schematic illustration in Figure 5E shows the suggested mechanism of how cell tension creates a pushing force against the rigid substrate on convex spherical geometries while a pulling force is created on concave ones – with a consequential impact on the nuclear morphology. Such a push‐pull mechanism was already presented by Delanoë‐Ayari and co‐workers for flat surfaces.^34^ It describes that tensile cytoskeletal (pull) forces pointing from the focal adhesions toward the cell's center create significant downward orientated compressive (push) forces at the nucleus.^34^, ^35^ On very soft materials this leads to a deformation of the substrate rather than of the nucleus (see^34^, ^35^ for detailed explanation). However, on materials that are significantly stiffer than the nucleus, as it is the case in our study (PTMC: 6 MPa, nucleus: 1–5 kPa^36^, ^37^), the substrate does not deform and the nucleus deforms under the vertical push‐force.

Thus, our data suggest that substrate curvature can induce changes in the 3D force patterns acting on the nucleus in adhering cells and thereby influence cellular differentiation. This mechanism seems to enable the cell to sense even small convex curvatures (curvature radii > cell size) and to discriminate between convex, flat and concave surfaces. It furthermore offers an explanation for the here observed induction of osteogenic differentiation on convex surfaces.

It has to be mentioned that other factors might influence cell behavior on differently curved surfaces. For example, cell adhesion is known to be influenced by the material's surface energy and the resulting surface tension at the liquid‐solid interface. The magnitude of surface curvature and the difference between concave, flat, and convex surfaces might additionally influence cell adhesion as a consequence of altered wettability. However, the highly comparable cell morphology that was observed on flat versus convex surfaces indicates that such effects are rather small and that the observed distinct attachment patterns are indeed mediated by the cytoskeletal‐tension and the resulting pull or push force. Furthermore, cytoskeletal push‐forces on convex surfaces might also be accompanied by an increase of hydrostatic pressure inside the cell therefore influencing cell behavior (e.g., signaling events) either directly or via the outflow of water and the reduction of cell volume.^38^, ^39^ Further studies are necessary to clarify the role of hydrostatic and osmotic pressure with respect to cellular response to curvature.

**Figure 6:**
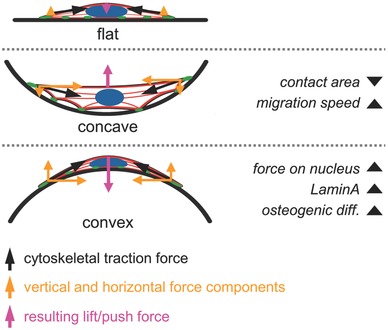
Schematic representation of the proposed geometry‐induced changes in cellular attachment and forces on the nucleus for flat, concave and convex surfaces. Nuclei are depicted in blue, F‐actin in red and focal adhesions in green. In cells on concave surfaces, cytoskeletal forces result in an upward pull force away from the substrate. The cell body is lifted away from the surface and remains attached only at distinct adhesion points while the nucleus is not exposed to severe cytoskeletal forces. In cells attached to convex spherical surfaces, cytoskeletal forces create a large push force toward the surface leading to compression and deformation of the nucleus. The mechanical forces on the nucleus induce high lamin‐A levels and promote osteogenic differentiation. As a consequence of the limited vertical extension of the cell, only a small push force is acting on the nucleus on flat surfaces.

The schematic representation in **Figure** **6** summarizes the proposed geometry‐induced changes in cellular attachment and nuclear response leading to increased lamin‐A levels on convex surfaces. These new insights are of relevance since 3D curvature is a tissue‐specific feature of the ECM but also an important material parameter in all porous biomaterials. A better understanding of the mechanisms involved in curvature‐sensation will help to implement curvature more successfully as a parameter to guide cell behavior in tissue regeneration.

## 3 Conclusions

In this study, we systematically investigated the influence of 3D surface curvature on cell and nucleus morphology and the subsequent effect on the migratory and differentiation behavior of hMSCs. Cells on concave spherical surfaces stretched upward, while cells on convex spherical surfaces were in full contact with the curved substrate and nuclei were flattened and deformed by the indenting actin cytoskeleton. Cells migrated significantly faster on concave spherical surfaces compared to flat and convex spherical surfaces as a consequence of a reduced contact between the cell and the material surface. In contrast, cells on convex surfaces showed a significantly higher intensity of the osteogenic marker osteocalcin indicating that cytoskeletal forces acting on the nucleus enhanced osteogenic differentiation. These observations indicate that the difference in cell attachment morphology caused by 3D substrate curvatures can modulate the cytoskeletal forces acting on the nucleus. 3D substrate curvature might thus be considered as a further cue to influence stem cell fate via mechanical control mechanisms. Based on these new insights of how geometrical signals influence cell behavior, our findings might contribute to a better understanding of mechanical control mechanisms in the interaction of cells with their extracellular environment. Such knowledge is expected to play an essential role in the development of future cell‐instructive biomaterial strategies. The incorporation of spatially controlled surface curvature as a material design parameter could provide an easy but potentially powerful tool for enhanced tissue regeneration.

## 4 Experimental Section

*Design and Production of the Cell Culture Chip*: Cell culture chips containing 3D structures were designed using computer‐aided design software (Rhinoceros 3D, McNeel Europe). The chips contained convex and concave spherical structures with principal curvatures of κ = 1/125, 1/175, 1/250, and 1/375 µm^−1^. Correct distance to the inverted microscope lens was ensured by 500 µm high feet at each corner and in the middle of the chip. Channels with diameters of 500 and 750 µm ensured sufficient medium exchange during imaging.

Three‐armed PTMC 5000 g mol^−1^ macromers were produced by ring‐opening polymerization of trimethylene carbonate (0.98 mol, 100 g, Huizhou Foryou Medical DevicesCo) initiated by tri(hydroxymethyl)propane (0.0196 mol, 2.62 g, Sigma‐Aldrich) and catalyzed by stannous octoate (Sn(Oct)_2_, 0.05 wt%, Sigma‐Aldrich) at a temperature of 130 °C for 3 d under argon atmosphere. Oligomers in solution in dry dichloromethane (100 mL) were end‐functionalized with methacrylate groups using methacrylic anhydride (0.176 mol, 26 mL, Sigma‐Aldrich) in the presence of triethylamine (0.176 mol, 25 mL, Sigma‐Aldrich) at room temperature for 5 d under argon atmosphere. Premature cross‐linking during the reaction was prevented by the addition of hydroquinone (0.06 wt%). Oligomers were subsequently precipitated in cold methanol. The degree of functionalization and molecular weight (Mn) of the macromer were determined by proton nuclear magnetic resonance (^1^H‐NMR, 300 MHz) analysis in deuterated chloroform (CDCl_3_). A photopolymerisable liquid resin was prepared by mixing PTMC macromers with 30 wt% propylene carbonate (Merck) as a diluent, 5 wt% Lucirin TPO‐L (BASF) as a photoinitiator, and 0.15 wt% Orasol Orange G dye (BASF) to control the cure depth of the light used for photopolymerization. Chips were built using a commercial stereolithography apparatus (Envisiontec Perfactory Mini Multilens SLA). The building process involved subsequent distinct pattern projections of 1280 × 1024 pixels, each 32 × 32 µm^2^ in size. Layers with a thickness of 15 µm were cured by irradiating for 45 s with blue light (400–550 nm). After the building process, uncured excess resin was extracted in propylene carbonate which was refreshed daily for one week. Then, the chips were washed during one week in gradients of ethanol/propylene carbonate mixtures to slowly shrink the chips to their final dimensions. Chips were finally dried in air. Chips were characterized by scanning electron microscopy (JCM‐6000; JEOL). The chips were sputter coated with gold (JFC‐1200; JEOL) and studied at a voltage of 5 kV.

*hMSC Isolation and Culture*: Bone marrow was obtained from human donors undergoing total hip joint replacements performed at Charité, Universitätsmedizin Berlin, Germany. This study was approved by the local ethical committee and all donors gave informed written consent. MSCs were isolated by density gradient separation using Histopaque‐1077 (Sigma‐Aldrich) and subsequent adhesion to tissue culture polystyrene. In this study, hMSCs from one donor (57y, m) were used at passage 2–5. Cells were cultured in expansion medium consisting of Dulbecco's modified Eagle's medium (Sigma‐Aldrich) supplemented with 10% fetal bovine serum (Biochrom AG), 1% penicillin/streptomycin (Biochrom AG), and 1% L‐glutamine (glutaMAX, Invitrogen). Medium was refreshed two times per week and the cells were trypsinized (PAA laboratories) when a confluency of 80% was reached.

*Migration Experiments*: hMSCs were stained with 10 × 10^−6^ m Cell Tracker Green (Life Technologies) and seeded on the chip. Time lapse imaging was performed using a Leica TCS SP 5 confocal microscope. An incubator chamber allowed live cell imaging at 37 °C and 5% CO_2_. Regions of interest were imaged with *z*‐stacks of 4 µm *z*‐spacing (≈40 planes per structure) at 512 × 512 pixels every 45 min for 24 h. Cell fluorescence was excited via the two‐photon laser at a wavelength of 910 nm. An automated *x*–*y* table allowed the imaging of multiple regions of interest per experiment. Migration experiments were repeated on six individual chips.

*Immunohistochemistry*: hMSCs were stained for osteocalcin after 10 d of culture in either expansion medium or osteogenic medium (expansion medium supplemented with 100 × 10^−9^ m Dexamethasone (Sigma‐Aldrich), 50 × 10^−6^ m Asorbic Acid (Sigma‐Aldrich), and 10 × 10^−3^ m β‐glycerol phosphate (Sigma‐Aldrich)). hMSCs were stained for vinculin and lamin‐A after 2 and 10 d of culture in expansion medium respectively. Cell seeded chips were fixated in 4% paraformaldehyde overnight and stained for osteocalcin (#13420, Abcam), vinculin (#V9131, Sigma‐Aldrich) or lamin‐A (#8980, Abcam). F‐actin was stained with Phalloidin 546 (in osteocalcin stained cells) or Phalloidin 633 (in vinculin and lamin‐A stained cells). Cell nuclei were counterstained with DAPI (Invitrogen). Immunohistologically stained chips were imaged using a Leica TCS SP5 microscope. A minimum of six concave/convex/flat structures from two chips were imaged per experimental group. *z*‐stacks were recorded at either 3 µm (osteocalcin) or 0.8 µm (vinculin and lamin‐A) *z*‐spacing at 2048 × 2048 pixels (vinculin) and 1024 × 1024 pixels (osteocalcin, lamin‐A). Laser power and detector settings were kept constant during the imaging of the different probes.

*Cell Speed Analysis*: Acquired time‐lapse images of the migration assay were analyzed using ImageJ plugin MtrackJ.^40^ The centers of in total 517 cells (164 cells on concave, 181 cells on convex, and 172 cells on flat surfaces) were tracked manually. The migration speed at each timeframe was calculated as the scalar of the displacement vector between two images, divided by the timeframe interval. Data from cells located at a *z*‐position within a range of 50 µm from the flat surface were excluded from further analysis. This criterion was chosen to analyze only cell movement on the structures and not on the surrounding flat surface or on the transition region from flat to curved surface. The average migration speed of every tracked cell on the structures was calculated.

*Data Analysis of Immunohistochemistry Data*: Images were analyzed using custom‐made macros in ImageJ to ensure a consistent analysis. For osteocalcin, F‐actin, and lamin‐A, *z*‐stack images were summed and plotted. Regions of interest were selected to exclude artefacts and information from surrounding flat surface (for convex/concave structures). Background fluorescence from the chip was subtracted. The average signal intensity per cell was calculated by multiplying the pixel intensity values with the number of pixels that contain this intensity, divided by the total cell number. For the vinculin images, the background was subtracted, images were binarized, and the number and areas of the focal adhesions were analyzed. Focal adhesions close to the nucleus (Figure 3C) were analyzed in a circular area of 405 µm^2^ around the center of the nucleus.

*Statistical Analysis*: The Kruskal‐Wallis test was used to assess differences between concave, convex, and flat surfaces and Dunn's test was performed for pairwise multiple comparison.

## Supporting information

As a service to our authors and readers, this journal provides supporting information supplied by the authors. Such materials are peer reviewed and may be re‐organized for online delivery, but are not copy‐edited or typeset. Technical support issues arising from supporting information (other than missing files) should be addressed to the authors.

Supplementary

Supplementary

Supplementary

Supplementary
