# Supplementary material for: Surface Curvature Differentially Regulates Stem Cell Migration and Differentiation via Altered Attachment Morphology and Nuclear Deformation
Source: Adv Sci (Weinh). 2016 Dec 20;4(2):1600347. doi: 10.1002/advs.201600347 (PMC5323878; doi:10.1002/advs.201600347)
Supplement: Supplementary file 1 — Supplementary [file ADVS-4-na-s001.pdf]

Copyright WILEY-VCH Verlag GmbH & Co. KGaA, 69469 Weinheim, Germany, 2016.

ADVANCED SCIENCE
Open Access

Supporting Information

for *Adv. Sci.,* DOI: 10.1002/advs.201600347

Surface Curvature Differentially Regulates Stem Cell Migration and Differentiation via Altered Attachment Morphology and Nuclear Deformation

*Maike Werner, Sébastien B. G. Blanquer, Suvi P. Haimi, Gabriela Korus, John W. C. Dunlop, Georg N. Duda, Dirk. W. Grijpma, and Ansgar Petersen**

Supporting Information

Figure S1: Mean hMSC migration speed on various curvature magnitudes. The mean hMSC migration speed over 24 hours on a flat surface and on concave and convex spherical surfaces of various curvature magnitudes. Mean +/- 95% CI.

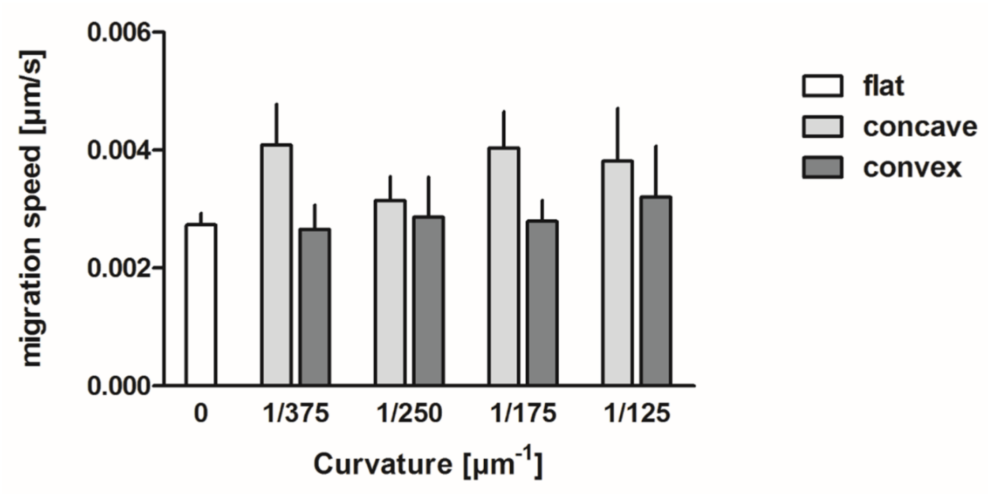

Movie S1: Time lapse recording of migrating hMSCs.

Movie S2: hMSCs adopt a spider-like morphology on concave spherical surfaces.

Movie S3: hMSCs adopt a snail-like morphology on convex spherical surfaces.
